# Relationships of Radiation Dose Indices with Body Size Indices in Adult Body Computed Tomography

**DOI:** 10.3390/tomography9040110

**Published:** 2023-07-14

**Authors:** Yusuke Inoue, Hiroyasu Itoh, Kazunori Nagahara, Hirofumi Hata, Kohei Mitsui

**Affiliations:** 1 Department of Diagnostic Radiology, Kitasato University School of Medicine, Sagamihara 252-0374, Japan; km19901004@gmail.com; 2 Department of Radiology, Kitasato University Hospital, Sagamihara 252-0375, Japan; hiroyasu@kitasato-u.ac.jp (H.I.); nagahara@kitasato-u.ac.jp (K.N.); kmri@kitasato-u.ac.jp (H.H.)

**Keywords:** computed tomography, radiation dose, body size, optimization

## Abstract

We investigated the relationships between radiation dose indices and body size indices in adult body computed tomography (CT). A total of 3200 CT scans of the thoracic, abdominal, abdominopelvic, or thoraco-abdominopelvic regions performed using one of four CT scanners were analyzed. Volume CT dose index (CTDIvol) and dose length product (DLP) were compared with various body size indices derived from CT images (water-equivalent diameter, WED; effective diameter, ED) and physical measurements (weight, weight/height, body mass index, and body surface area). CTDIvol showed excellent positive linear correlations with WED and ED. CTDIvol also showed high linear correlations with physical measurement-based indices, whereas the correlation coefficients were lower than for WED and ED. Among the physical measurement-based indices, weight/height showed the strongest correlations, followed by weight. Compared to CTDIvol, the correlation coefficients with DLP tended to be lower for WED, ED, and weight/height and higher for weight. The standard CTDIvol values at 60 kg and dose increase ratios with increasing weight, estimated using the regression equations, differed among scanners. Radiation dose indices closely correlated with body size indices such as WED, ED, weight/height, and weight. The relationships between dose and body size differed among scanners, indicating the significance of dose management considering body size.

## 1. Introduction

Computed tomography (CT) delivers relatively high radiation doses to patients, which makes the risk of inducing cancer a major concern when using CT in clinical medicine [1,2,3]. Radiation dose management is essential to reduce radiation dose while maintaining appropriate image quality and diagnostic performance. Volume CT dose index (CTDIvol) and dose length product (DLP) are commonly used as radiation dose indices in CT [4,5]. They are automatically provided by the CT scanner during each examination. CTDIvol represents radiation exposure to the scan region and is calculated based on imaging parameters such as tube current, rotation time, and pitch and the absorbed dose measured using a dosimetry phantom. DLP is an integral of CTDIvol over the entire scan range and represents the total radiation exposure in an examination. Effective dose, representing the risk of stochastic effects such as cancer induction and hereditary effects, may be calculated by multiplying DLP by an appropriate conversion factor [4]. Size-specific dose estimate is a relatively new dose index defined as CTDIvol corrected for the cross-sectional size. It is considered to reflect the patient’s absorbed dose more accurately than CTDIvol [6]; however, the scanner does not provide it automatically. Therefore, CTDIvol is commonly currently used in radiation dose management. The use of diagnostic reference levels (DRLs) is recommended to optimize the radiation dose in medical imaging. The DRLs for CT are usually established using CTDIvol and DLP as indices of radiation dose [7].

With increasing body size, X-rays are attenuated more severely while passing the body. As a result, the number of X-ray photons reaching the detector decreases at a given amount of X-ray exposure, increasing image noise. Therefore, increased radiation exposure is required to preserve the image quality in a large patient. The X-ray output is proportional to the tube current. Automatic exposure control (AEC) assesses the strength of attenuation of the image section mainly based on the localizer image and automatically modulates the tube current [8,9,10]. AEC adjusts radiation exposure for each patient and position according to the attenuation strength and contributes to optimization of the radiation dose.

Because the required radiation exposure varies depending on the body size, considering body size is critical in radiation dose management [11]. Although DRLs are established for weight- or age-based groups in pediatric CT [7,12], they are usually determined only for standard-sized patients in adult CT. Each facility compares the radiation dose used at the facility to the DRLs based on data from examinations performed in standard-sized patients. However, the type of AEC software and parameter settings affect the resulting tube current modulation [13,14,15,16] and the effect of body size on the modulation [16,17,18]. Therefore, radiation dose should be evaluated in various body sizes, and DRLs according to body size have been reported in adult CT [19,20].

Positive correlations between CT radiation dose and body size indices, such as body weight and body mass index (BMI), have been reported [17,18,21,22,23,24]. Besides such indices based on physical measurements, correlations with CT radiation dose have been reported for effective diameter (ED) and water-equivalent diameter (WED), body size indices based on images [24,25,26,27,28]. ED is typically defined as a geometric mean of the anteroposterior and lateral diameters [6] and is calculated from the localizer or CT images. WED is a more sophisticated index considering the differences in attenuation strength between tissues and is calculated from CT images [29]. Image-based indices represent the degree of attenuation in the image section more accurately than physical measurement-based indices; however, image analysis is required to obtain them. Physical measurement-based indices are more convenient and can be used to predict the radiation dose for a given patient before an examination.

For radiation dose management considering body size, patients are usually grouped according to body size indices in both children [7,12] and adults [19,20,30], while dose management regarding the body size indices as continuous variables has also been reported in children [31,32] and adults [18,26]. This study investigated the relationships of CT radiation dose indices with various body size indices regarded as continuous variables in the context of adult body CT. We aimed to improve the method of radiation dose management considering body size in adult body CT.

## 2. Materials and Methods

### 2.1. Subjects

Thoracic, abdominal, abdominopelvic, or thoraco-abdominopelvic CT scans with or without contrast enhancement performed according to our standard protocol were retrospectively analyzed. Four CT scanners were used, and 200 CT scans (100 scans each in females and males) were consecutively enrolled for each scan region and scanner; 3200 scans were included. The exclusion criteria were as follows: age < 20 years, imaging without raising the arms, body weight > 100 kg, body height < 130 cm, and BMI > 40 kg/m^2^. When two or more series of CT images were acquired in an examination, a single series was used for analysis. Kitasato University Medical Ethics Organization (Sagamihara, Japan) approved this study (B22-166). Due to the retrospective nature of the study, the need for informed consent was waived.

### 2.2. Imaging Procedures

The four CT scanners were SOMATOM Definition Flash (Siemens, Erlangen, Germany), Optima CT 660 Discovery Edition (GE Healthcare, Milwaukee, WI, USA), LightSpeed VCT VISION (GE Healthcare, Milwaukee, WI, USA), and SCENARIA (Fujifilm Healthcare Corp., Tokyo, Japan). They were named as Siemens, GEa, GEb, and Fujifilm scanners, respectively.

Tube current was modulated using AEC. CARE Dose 4D was used for the Siemens scanner, with a quality reference mAs of 180 mAs (thoracic CT) or 240 mAs (the other three scan regions) and a modulation strength of average (slim)/average (obese). The organ setting was the thorax for thoracic CT and the abdomen for the other three scan regions. When an anthropomorphic phantom was imaged, the tube current modulation curve obtained with a quality reference mAs of 180 mAs and the thorax setting was similar to that obtained with a quality reference mAs of 240 mAs and the abdomen setting. Two localizer images in the posteroanterior and lateral directions were used to estimate the attenuation strength.

For the GEa and GEb scanners, Auto mA and Smart mA were used with a noise index of 17, maximum current of 350 mA (thoracic CT) or 400 mA (the other three scan regions), and minimum current of 50 mA. A lateral localizer image was used to estimate the attenuation strength. In addition, organ dose modulation was applied in thoracic and thoraco-abdominopelvic CT for the GEa scanner, and tube current from the anterior side was reduced for the thyroid in all patients and for the breast in females.

Intelli EC was used for the Fujifilm scanner with a noise index of 17.5, maximum current of 350 mA (thoracic CT) or 420 mA (the other three scan regions), and minimum current of 50 mA. A lateral localizer image was used to estimate the attenuation strength.

The tube voltage was 120 kV for all scanners. The beam width was 38.4 mm for the Siemens scanner and 40 mm for the GEa, GEb, and Fujifilm scanners. Rotation time was 0.5 s/rotation for the Siemens, GEa, and GEb scanners and 0.4 s/rotation for the Fujifilm scanner. The pitch was 1.2, 0.984, 0.984, and 0.8281 for the Siemens, GEa, GEb, and Fujifilm scanners, respectively.

The images were reconstructed with hybrid reconstruction techniques: sinogram-affirmed iterative reconstruction (SAFIRE, level 1) for the Siemens scanner, adaptive statistical iterative reconstruction (ASiR, 50% IR blending) for the GEa and GEb scanners, and Intelli IP (level 3) for the Fujifilm scanner. Standard images for the soft-tissue display were reconstructed with a slice thickness of 3 mm (Siemens scanner) or 2.5 mm (GEa, GEb, and Fujifilm scanners) and a slice increment of 2.5 mm. Lung images were also reconstructed in thoracic and thoraco-abdominopelvic CT.

### 2.3. Data Analysis

CTDIvol and DLP were calculated by the CT scanner and used as the radiation dose indices. Body height and body weight were recorded at the time of CT. The body size indices used for the analysis were two image-based indices—WED and ED—and four physical measurement-based indices—weight, weight/height, BMI, and body surface area (BSA). WED and ED were determined based on CT images using the dose management system (Radimetrics; Bayer Medical Care Inc., Indianola, PA, USA). The mean value for the entire scan range was used for analysis. The BSA (m^2^) was calculated as 0.024265 × W^0.5378^ × H^0.3964^ [33], where W is weight in kg and H is height in cm.

CTDIvol and DLP were plotted against the body size indices, and linear regression analysis was performed with the least-squares method to determine a linear regression equation and correlation coefficient. The standard dose at a weight of 60 kg was estimated by substituting a weight value of 60 kg into the regression equation between weight and dose. Moreover, the dose increase ratio, representing the degree of dose increase with weight increase from 50 to 70 kg, was calculated as follows:

dose increase ratio = (D70 − D50)/D60 × 100

where D50, D60, and D70 are standard doses at 50, 60, and 70 kg, respectively, estimated using the regression equations.

## 3. Results

### 3.1. Relationships of CTDIvol with Body Size Indices

**Figure 1:**
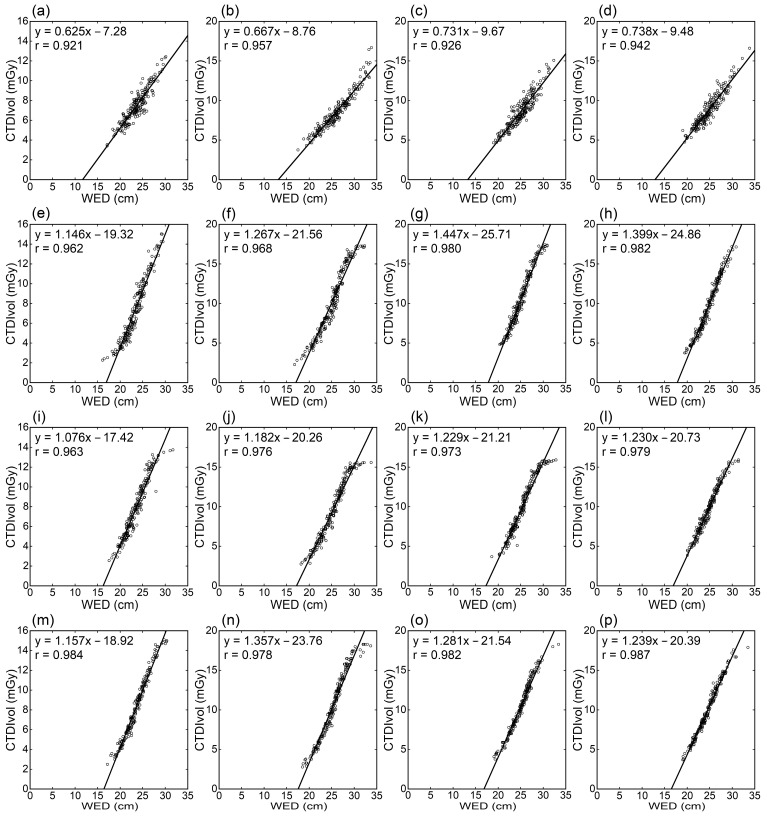
Relationships between CTDIvol and WED. Panels (**a**–**d**), (**e**–**h**), (**i**–**l**), and (**m**–**p**) are for the Siemens, GEa, GEb, and Fujifilm scanners, respectively. Results of thoracic (**a**,**e**,**i**,**m**), abdominal (**b**,**f**,**j**,**n**), abdominopelvic (**c**,**g**,**k**,**o**), and thoraco-abdominopelvic CTs (**d**,**h**,**l**,**p**) are presented.

CTDIvol increased with increasing WED, regardless of the scan region or CT scanner (Figure 1). Excellent linear correlations were demonstrated between CTDIvol and WED. However, some plots were apparently below the regression line at the right end, corresponding to the largest WED, for the GEb and Fujifilm scanners. The relationship was similar among the GEa, GEb, and Fujifilm scanners but mildly different for the Siemens scanner. Compared to the Siemens scanner, the slopes of the regression lines were larger and the y-intercept was smaller for the other three scanners, indicating a more prominent CTDIvol increase with increasing WED. The correlation coefficients were 0.921–0.957 for the Siemens scanner and were higher for the other three scanners (GEa, 0.962–0.982; GEb, 0.963–0.979; Fujifilm, 0.978–0.987) (Figure 1, Table 1).

CTDIvol also showed excellent positive linear correlations with ED. The correlation coefficients were lower for ED than for WED; however, the differences were small (Table 1).

**Figure 2:**
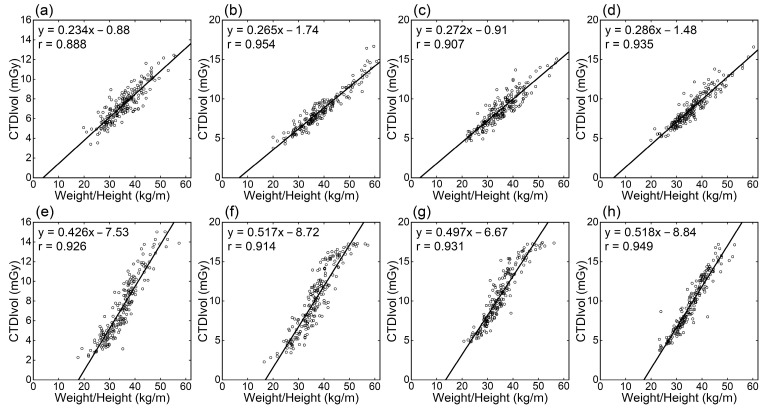
Relationships between CTDIvol and weight/height. Panels (**a**–**d**) and (**e**–**h**) are for the Siemens and GEa scanners, respectively. Results of thoracic (**a**,**e**), abdominal (**b**,**f**), abdominopelvic (**c**,**g**), and thoraco-abdominopelvic CTs (**d**,**h**) are presented.

**Figure 3:**
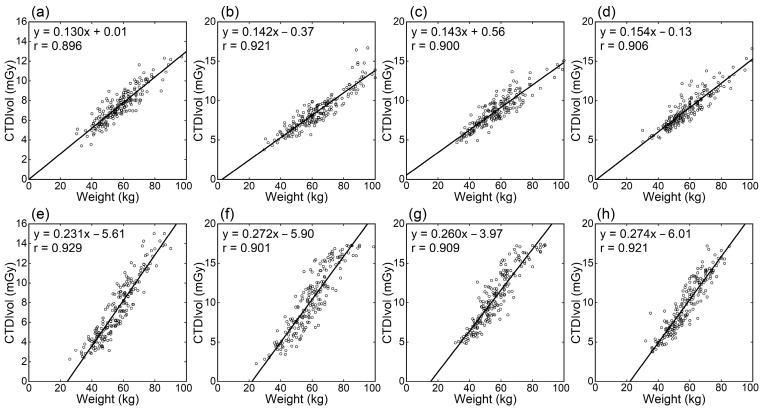
Relationships between CTDIvol and weight. Panels (**a**–**d**) and (**e**–**h**) are for the Siemens and GEa scanners, respectively. Results of thoracic (**a**,**e**), abdominal (**b**,**f**), abdominopelvic (**c**,**g**), and thoraco-abdominopelvic CTs (**d**,**h**) are presented.

CTDIvol generally showed high positive linear correlations with the physical measurement-based indices of body size (weight, weight/height, BMI, and BSA), whereas the correlation coefficients were lower than for WED and ED (Table 1). Among physical measurement-based indices, the correlation tended to be strongest for weight/height (Figure 2), followed by weight (Figure 3). The correlation was weaker for BMI and BSA. The correlation coefficients did not differ notably between scanners.

### 3.2. Relationships of DLP with Body Size Indices

The correlation coefficients between DLP and various body size indices are presented in Table 2. Compared to the correlation coefficients with CTDIvol, those with DLP tended to be lower for WED, ED, weight/height, and BMI, and higher for weight and BSA. DLP showed strong correlations with WED and ED; however, the superiority of WED and ED over weight was less apparent for DLP than for CTDIvol. The superiority of weight/height over weight, observed for CTDIvol, disappeared for DLP. The correlation coefficients between DLP and weight were especially high in thoraco-abdominopelvic CT.

The standard radiation doses at 60 kg calculated using the linear regression equations are presented in Table 3, and the dose increase ratios are presented in Table 4. The standard CTDIvol at 60 kg differed mildly between the scan regions; it was high for abdominopelvic CT, lower for abdominal CT, and even lower for thoracic CT. The standard dose at 60 kg was lower for the Siemens scanner than for the other scanners. The dose increase ratio was lower for the Siemens scanner than for the other scanners. The dose increase ratio was slightly higher for DLP than for CTDIvol. When comparing the two GE scanners, the standard dose at 60 kg was lower in abdominal and abdominopelvic CT for the GEb scanner than for the GEa scanner but similar in thoracic CT between the two scanners.

## 4. Discussion

This study investigated the relationships between radiation dose indices and body size indices in adult body CT. Four CT scanners from three manufacturers were used, and four scan regions were analyzed. Two image-based indices (WED and ED) and four physical measurement-based indices (weight, weight/height, BMI, and BSA) were examined as body size indices. These indices were regarded as continuous variables when compared with dose indices.

CTDIvol increased with increasing WED regardless of the scan region or CT scanner, and regression lines were successfully fitted to the plots of CTDIvol against WED. These results indicated the effectiveness of linear regression between CTDIvol and WED in radiation dose management considering body size. In addition, since WED represents the attenuation strength of an image section, the excellent correlation between CTDIvol and WED implies that AEC achieved appropriate radiation dose modulation according to the attenuation strength in adult body CT covering various scan ranges. Some examinations performed on the GEb and Fujifilm scanners showed apparently lower CTDIvol than predicted from the linear relationships and WED. A maximum mA is set when using AEC software installed on the GE and Fujifilm scanners. In this study, a tube current equal to the maximum mA value was applied to most or all image slices in very large patients. The maximum setting is indicated to have prevented the scanner from applying a higher tube current, which appears responsible for the lower CTDIvol than predicted.

The relationship between CTDIvol and WED for the Siemens scanner differed from those for the other three scanners. The increase in CTDIvol with increasing WED was milder than for the other scanners, in line with previous studies [16,17,18]. AEC systems in the GE and Fujifilm scanners modulate tube current to maintain the noise level of CT images reconstructed by the standard method. For the Siemens scanner, the degree of increase in tube current with increasing attenuation is selectable and adjusted by the modulation strength [14,15]. In this study, average modulation was used; it is assumed that selecting strong or very strong modulation would reduce the differences between the Siemens scanner and the other scanners. The relationship between radiation dose and body size depends on the CT scanner and AEC settings. Our results confirm the significance of evaluating radiation doses not only in standard-sized patients but also in those with various body sizes.

Besides WED, ED was assessed as an image-based index of attenuation strength. Whereas WED is a sophisticated index that considers differences in attenuation among tissues, ED is a simple index of cross-section size and is easy to calculate. Although the correlation with CTDIvol was weaker for ED than for WED, the difference was small, and the use of ED is considered acceptable when WED is not readily available.

The WED and ED directly reflect the attenuation strength of the image section but require data processing using localizer or CT images. Alternatively, simpler indices based on physical measurements, such as weight or indices calculated from height and weight, may be used. Using physical measurement-based indices, the radiation dose can be predicted before CT imaging. This study examined the relationships of weight, weight/height, BMI, and BSA with radiation dose indices. Weight/height showed the strongest correlation with CTDIvol, followed by weight; correlations were weaker for BMI and BSA. A height increase with a fixed cross-sectional size will increase weight but not CTDIvol. Differences in height appear to weaken the correlation between weight and CTDIvol. It is indicated that weight/height is more suitable for radiation dose management using CTDIvol since weight/height reflects cross-sectional size better than weight. However, height is often not recorded in clinical practice. The differences in the correlations of CTDIvol with weight and weight/height were small. Therefore, weight-based dose management appears to be acceptable where height records are unavailable.

In this study, the correlation coefficients with DLP tended to be lower for WED, ED, weight/height, and BMI, and higher for weight and BSA, than those with CTDIvol. Whereas DLP increases with increasing scan length, WED, ED, weight/height, and BMI appear to be indicators of cross-sectional attenuation strength. Variations in scan length in patients with the same attenuation strength appear to weaken the correlation between these indices and DLP. In contrast, weight and BSA are indices of overall body size and increase with height even when the cross-sectional size is constant. Higher correlation coefficients with DLP than with CTDIvol for weight and BSA appear to be attributable to the effect of prolongation of the scan length with increasing height. Weight and weight/height are equivalent as body size indices for radiation dose management using DLP, and weight is preferable because of its simplicity. On the other hand, although WED and ED represent cross-sectional sizes, they still correlate closely with DLP and are suitable for radiation dose management using DLP.

The distribution of body mass differs in patients with the same weight, leading to differences in radiation dose for thoracic, abdominal, and abdominopelvic CT. For example, in a patient with a small thorax and a large pelvis, the radiation dose will be low in thoracic CT and high in abdominopelvic CT. The effects of the distribution on radiation dose is counterbalanced in thoraco-abdominopelvic CT, which appears to have caused especially high correlation coefficients between DLP and weight.

Determining a regression equation between the body size index as a continuous variable and the dose index allows the calculation of a standard dose index at an arbitrary value of the body size index. A table presenting dose indices for representative body sizes facilitates comparison of doses between scanners or scan regions. The standard radiation dose estimated at 60 kg was lower for the Siemens scanner than for the other scanners at our facility. The reconstruction algorithm differed among manufacturers, and the slice was thicker for the Siemens scanner (3 mm) than for the other scanners (2.5 mm). Our reconstruction conditions for the Siemens scanner were assumed to have more robust noise reduction properties. The standard dose was lower in abdominal and abdominopelvic CT using the GEb scanner than the GEa scanner. This difference is because the GEb scanner delivers a lower dose at the same tube current than the GEa scanner when measured using a dosimetry phantom. It should be noted that such differences may exist even between scanners provided by the same manufacturer. For thoracic CT, the doses were similar for the two GE scanners, presumably due to the use of organ dose modulation in the GEa system. This function decreases radiation exposure from the anterior direction and does not change exposure from the posterior direction, reducing the overall dose [34,35].

We compared the dose increase ratio with increasing weight around 60 kg. There were large differences in the ratios between scanners, supporting the significance of evaluating doses not only for standard-sized patients but also those with various body sizes. The dose increase ratio was slightly higher for DLP than for CTDIvol, presumably due to the prolongation of the scan length with increasing overall body size.

Radiation dose management using regression equations between dose indices and body size indices has expected advantages besides evaluating the standard dose and its dependence on body size for each scanner and imaging protocol. Routine dose monitoring should be conducted to identify examinations with exceptionally high doses and investigate their validities and causes [11,18,26,27,30]. If high-dose examinations are extracted simply based on the absolute values of CTDIvol or DLP, most extracted examinations would be those performed in large patients [18]. A high dose in a large patient may be necessary and does not imply an excessive dose. The standard dose can be estimated for each examination using the regression equation between the dose index and body size index. By comparing the actual recorded dose with the estimated dose, the excessive dose events to be investigated can be effectively and efficiently identified [11,18]. In addition, when using a new scanner or new imaging protocol, it is desirable to evaluate dose adequacy early using small amounts of examination data. If only examinations performed in standard-sized patients are selected, collecting sufficient data for meaningful dose evaluation may take a long time. Analysis of dose indices combined with body size indices using all examinations would allow early assessment of the validity of new imaging conditions. In this study, the usefulness of the body size index was investigated based on the degree of correlation with the dose index, as well as availability. A high correlation is expected to increase the detection sensitivity of excessive dose events and decrease uncertainty in regression equations determined using a small amount of data.

This study had some limitations. Diagnostic performance should be considered together with radiation dose for optimization. Data used in this study were collected from our routine clinical practice, and the imaging protocols were considered acceptable in terms of radiation dose and image quality. However, no systematic evaluation of image quality was performed in this study. Investigation of dose–size relationships combined with image quality evaluation remains to be done. The AEC parameter settings are determined at each facility and influence the relationship between dose and body size. The effects of AEC settings on the relationship should be studied in the future. This study only used examinations performed according to the standard protocol at our facility to determine the standard radiation dose and its relationship with body size. Variances from the protocol are infrequent and recorded during daily clinical practice at our facility, which may differ from other facilities. The degree of correlations between the dose index and body size index would vary when many variant examinations are included in analysis. Data used in this study were obtained from Japanese patients. The size and shape of the body would differ among countries, and evaluation in other counties is desired. Although weight was measured for enhanced CT just before the examination, weight records for unenhanced CT and height records were mainly based on medical interviews or previous records in the electronic medical system. Therefore, their accuracies were not guaranteed.

## 5. Conclusions

This study investigated the relationships between radiation dose indices and various body size indices in adult body CT. The correlations between WED and CTDIvol were excellent, confirming that AEC achieves dose modulation according to differences in body size among patients. The relationship between dose and body size differed among CT scanners, indicating the significance of radiation dose management considering body size. Among physical examination-based indices, weight/height and weight are suitable for dose management.

## Figures and Tables

**Table 1:** Correlation coefficients between CTDIvol and body size indices.

Region	Scanner	Correlation Coefficients					
		WED (cm)	ED (cm)	Weight (kg)	W/H (kg/m)	BMI (kg/m^2^)	BSA (m^2^)
T	Siemens	0.921	0.913	0.896	0.888	0.799	0.873
	GEa	0.962	0.952	0.929	0.926	0.846	0.901
	GEb	0.963	0.950	0.889	0.907	0.821	0.853
	Fujifilm	0.984	0.961	0.902	0.923	0.875	0.861
A	Siemens	0.957	0.955	0.921	0.954	0.928	0.871
	GEa	0.968	0.966	0.901	0.914	0.854	0.873
	GEb	0.976	0.971	0.908	0.918	0.865	0.885
	Fujifilm	0.978	0.967	0.889	0.898	0.842	0.864
AP	Siemens	0.926	0.919	0.900	0.907	0.838	0.877
	GEa	0.980	0.976	0.909	0.931	0.883	0.877
	GEb	0.973	0.966	0.909	0.927	0.881	0.883
	Fujifilm	0.982	0.976	0.922	0.953	0.914	0.881
TAP	Siemens	0.942	0.940	0.906	0.935	0.895	0.859
	GEa	0.982	0.975	0.921	0.949	0.883	0.871
	GEb	0.979	0.972	0.905	0.943	0.898	0.854
	Fujifilm	0.987	0.980	0.918	0.949	0.898	0.879
T	All	0.957	0.944	0.904	0.911	0.836	0.872
		(0.026)	(0.021)	(0.017)	(0.018)	(0.033)	(0.021)
A	All	0.970	(0.965	(0.905	0.921	0.872	0.873
		(0.009)	(0.007)	(0.013)	(0.023)	(0.038)	(0.009)
AP	All	0.965	0.959	(0.910	0.930	0.879	0.880
		(0.026	(0.027	(0.009	(0.019	(0.031	(0.003
TAP	All	0.973	0.967	0.913	0.944	0.894	0.866
		(0.021)	(0.018)	(0.008)	(0.007)	(0.007)	(0.011)
All	Siemens	0.937	0.932	0.906	0.921	0.865	0.870
		(0.016)	(0.019)	(0.011)	(0.029)	(0.058)	(0.008)
All	GEa	0.973	0.967	0.915	0.930	0.867	0.880
		(0.010)	(0.011)	(0.012)	(0.014)	(0.019)	(0.014)
All	GEb	0.972	0.965	0.903	0.924	0.866	0.869
		(0.007)	(0.010)	(0.009)	(0.015)	(0.033)	(0.018)
All	Fujifilm	0.983	0.971	0.908	0.931	0.882	0.871
		(0.004)	(0.009)	(0.015)	(0.026)	(0.031)	(0.010)
All	All	0.966	0.959	0.908	0.926	0.870	0.873
		(0.020)	(0.020)	(0.012)	(0.020)	(0.035)	(0.013)

W/H = weight/height, T = thoracic CT, A = abdominal CT, AP = abdominopelvic CT, TAP = thoraco-abdominopelvic CT. All for scanner indicates the mean (SD) for four scanners. All for region indicates the mean (SD) for four regions. “All” for both region and scanner indicates the mean (SD) of 16 correlation coefficients.

**Table 2:** Correlation coefficients between DLP and body size indices.

Region	Scanner	Correlation Coefficients					
		WED (cm)	ED (cm)	Weight (kg)	W/H (kg/m)	BMI (kg/m^2^)	BSA (m^2^)
T	Siemens	0.819	0.847	0.893	0.827	0.676	0.902
	GEa	0.933	0.941	0.938	0.912	0.806	0.924
	GEb	0.914	0.938	0.923	0.896	0.757	0.910
	Fujifilm	0.959	0.954	0.912	0.902	0.819	0.890
A	Siemens	0.945	0.933	0.919	0.938	0.897	0.876
	GEa	0.950	0.944	0.896	0.906	0.842	0.866
	GEb	0.959	0.947	0.906	0.910	0.852	0.883
	Fujifilm	0.962	0.945	0.884	0.884	0.818	0.861
AP	Siemens	0.922	0.913	0.922	0.911	0.819	0.904
	GEa	0.969	0.961	0.919	0.926	0.860	0.892
	GEb	0.969	0.961	0.916	0.921	0.859	0.894
	Fujifilm	0.968	0.959	0.930	0.946	0.889	0.895
TAP	Siemens	0.921	0.919	0.937	0.923	0.834	0.914
	GEa	0.964	0.959	0.951	0.942	0.834	0.919
	GEb	0.965	0.961	0.939	0.943	0.858	0.906
	Fujifilm	0.975	0.969	0.951	0.944	0.846	0.930
T	All	0.906	0.920	0.917	0.884	0.765	0.907
		(0.061)	(0.049)	(0.019)	(0.039)	(0.065)	(0.014)
A	All	0.954	0.942	0.901	0.909	0.852	0.871
		(0.008)	(0.006)	(0.015)	(0.022)	(0.033)	(0.010)
AP	All	0.957	0.948	0.922	0.926	0.857	0.896
		(0.023)	(0.024)	(0.006)	(0.015)	(0.029)	(0.005)
TAP	All	0.956	0.952	0.945	0.938	0.843	0.917
		(0.024)	(0.022)	(0.007)	(0.010)	(0.012)	(0.010)
All	Siemens	0.902	0.903	0.918	0.900	0.806	0.899
		(0.056)	(0.038)	(0.018)	(0.050)	(0.093)	(0.016)
All	GEa	0.954	0.951	0.926	0.922	0.835	0.900
		(0.016)	(0.010)	(0.024)	(0.016)	(0.022)	(0.027)
All	GEb	0.952	0.951	0.921	0.917	0.832	0.898
		(0.025)	(0.011)	(0.014)	(0.020)	(0.050)	(0.012)
All	Fujifilm	0.966	0.957	0.919	0.919	0.843	0.894
		(0.007)	(0.010)	(0.028)	(0.031)	(0.033)	(0.028)
All	All	0.943	0.941	0.921	0.914	0.829	0.898
		(0.038)	(0.029)	(0.020)	(0.030)	(0.052)	(0.020)

W/H = weight/height, T = thoracic CT, A = abdominal CT, AP = abdominopelvic CT, TAP = thoraco-abdominopelvic CT. “All” for scanner indicates the mean (SD) for four scanners. “All” for region indicates the mean (SD) for four regions. “All” for both region and scanner indicates the mean (SD) of 16 correlation coefficients. 3.3. Standard radiation doses and dose increase ratios.

**Table 3:** Standard radiation doses at 60 kg.

Scanner	CTDI at 60 kg (mGy)				DLP at 60 kg (mGy∙cm)			
	T	A	AP	TAP	T	A	AP	TAP
Siemens	7.81	8.16	9.14	9.13	276.6	217.4	437.8	616.6
GEa	8.25	10.45	11.62	10.42	308.3	297.4	576.6	714.6
GEb	8.45	9.48	10.57	10.35	311.1	269.8	526.3	707.6
Fujifilm	9.03	10.32	11.46	10.79	331.2	294.7	571.7	747.2

T = thoracic CT, A = abdominal CT, AP = abdominopelvic CT, TAP = thoraco-abdominopelvic CT.

**Table 4:** Dose increase ratios.

Scanner	Increase Ratio of CTDIvol				Increase Ratio of DLP			
	T	A	AP	TAP	T	A	AP	TAP
Siemens	33.3	34.9	31.3	33.8	35.4	44.5	38.9	37.7
GEa	56.0	52.1	44.7	52.6	57.9	59.2	50.8	56.8
GEb	47.2	52.8	44.4	46.8	50.3	59.5	51.0	50.7
Fujifilm	51.1	55.6	44.5	44.4	51.9	63.4	50.7	48.8

T = thoracic CT, A = abdominal CT, AP = abdominopelvic CT, TAP = thoraco-abdominopelvic CT.

## Data Availability

The data are available upon reasonable request from the corresponding author.
